# Patterns of wasting among pregnant and lactating women in Uganda, 2015–2018: analysis of Nutrition surveillance data

**DOI:** 10.1186/s40795-021-00464-w

**Published:** 2021-10-14

**Authors:** Irene Byakatonda Kyamwine, Samalie Namukose, Yvette Wibabara, Lilian Bulage, Benon Kwesiga, Alex Riolexus Ario, Julie R. Harris

**Affiliations:** 1 Uganda Public Health Fellowship Program, Ministry of Health, Kampala, Uganda; 2 Nutrition Division, Ministry of Health, Kampala, Uganda; 3 Workforce and Institute Development Branch, Division of Global Health Protection, Center for Global Health, US Centers for Disease Control and Prevention, Kampala, Uganda

**Keywords:** Wasting, Pregnant, Lactating, Women, Uganda

## Abstract

**Background:**

Maternal nutrition is closely linked to the survival and development of children during the first 1000 days of life. Maternal wasting, a measure of malnutrition, is measured using the mid-upper arm circumference. However, in 2019, the rate and distribution of wasting among pregnant and lactating women was not known. We described annual trends and distribution of wasting among pregnant and lactating women (PLW), Uganda, 2015–2018, to inform programming on targeted nutritional interventions.

**Methods:**

We analyzed nutrition surveillance data from the District Health Information System for all PLW from 2015 to 2018. We used the World Health Organization standard thresholds to determine wasting among PLW by year and region, drawing choropleth maps to demonstrate the geographic distribution of wasting among PLW. We used logistic regression to assess wasting trends.

**Results:**

During 2015–2018, 268,636 PLW were wasted (prevalence = 5.5%). Of the 15 regions of Uganda, Karamoja (prevalence = 21%) and Lango (prevalence = 17%) registered the highest prevalence while Toro (prevalence = 2.7%) and Kigezi (prevalence = 2.0%) registered the lowest prevalence. The national annual prevalence of wasting among PLW declined by 31% from 2015 to 2018 (OR = 0.69, *p* < 0.001). Regions in the north had increasing trends of wasting over the period [Lango (OR = 1.6, *p* < 0.001) and Acholi (OR = 1.2, *p* < 0.001)], as did regions in the east [(Bugisu (OR = 3.4, *p* < 0.001), Bukedi (OR = 1.4, *p* < 0.001), and Busoga (OR = 1.3, *p* < 0.001)]. The other 11 regions showed declines.

**Conclusion:**

The trend of wasting among PLW nationally declined during the study period. Lango and Acholi regions, both of which were experiencing a nutrition state of emergency during this period, had both high and rising rates of wasting, as did the Karamoja region, which experienced the highest wasting rates. We recommended that the Ministry of Health increases its focus on nutrition monitoring for PLW and conduct an analysis to clearly identify the factors underlying malnutrition specific for PLW in these regions.

## Background

Malnutrition refers to deficiencies, excesses, or imbalances in a person’s intake of energy and/or nutrients. It includes both undernutrition, which covers stunting, wasting, and micronutrient deficiencies, and overnutrition [1]. Wasting refers to low weight-for-height and usually indicates a recent weight loss; if not addressed promptly, wasting is often associated with morbidity and mortality among both adults and children [2, 3].

Adequate nutrition is crucial for the survival, health, and development of mothers and their children [4, 5]. During pregnancy and lactation, there is an increased demand for energy, protein, and essential micronutrients to maintain not only the mother’s but also the child’s health and development [6, 7]. Maternal malnutrition predisposes mothers to maternal complications, and children to fetal birth defects, low birth weight, restricted physical and mental potential, and fetal or newborn mortality [8] and accounts for approximately 20% of childhood stunting [9]. A malnourished mother is more likely to deliver a malnourished baby, who will grow into a malnourished adult [8]. Because of this, ending malnutrition among pregnant and lactating women is a critical factor in breaking the cycle of malnutrition in the population.

Despite a decline in the prevalence of malnutrition globally, undernutrition remains a challenge of public health concern worldwide. In 2016, the global prevalence of underweight (9.7%) and anemia (32.8%) among women of reproductive age (15–49 years) were still above the World Health Assembly (WHA) 2025 targets of 5% for underweight and 15% for anemia [10–12]. In Uganda in 2016, the prevalence of underweight and anemia among women of reproductive age were 9 and 32%, respectively [13, 14].

In response to the National Development Plan (2010–2015) objective of improved nutrition in the Ugandan population, Uganda began scaling up the Nutrition Strategy with a focus on the first 1000 days of life, which refers to the period of time (for both the mother and the fetus/infant) from conception to 2 years of age [15, 16]. In 2011, Uganda developed a national nutrition strategy (Uganda Nutrition Action Plan 1, or UNAP 1) aimed at breaking the cycle of malnutrition and improving the livelihood of Ugandans [16].

During the 5 years of implementing the strategy (2011–2016), the prevalence of thinness (wasting) among non-pregnant or lactating women of reproductive age in Uganda declined from 11 to 9%. During the same period, wasting among children also declined from 5 to 4% and stunting declined from 33 to 29% [13]. However, wasting specific to pregnant or lactating women (PLW) in Uganda has not yet been evaluated. Since the strategy focuses on the first 1000 days of life understanding nutrition state of pregnant and lactating women who make up the largest part of this period is crucial for programing. We estimated the prevalence and described the trends and geographical distribution of wasting among PLW in Uganda during 2015–2018 to inform targeted programming to break the cycle of malnutrition.

## Methods

### Study setting

Our study utilized data collected from all the 15 subregions of Uganda, with 135 districts [17, 18]. In 2019, Uganda had an estimated population of 40 million persons [19].

### Study design

We conducted a retrospective descriptive analysis of nutrition surveillance data from the District Health Information Software 2 (DHIS2), which are submitted quarterly from all the health facilities in Uganda. Our study focused on wasting because it accounts for higher proportions of intrauterine growth retardation (IUGR) and impaired fetal development, which carry lifelong adverse effects in children [5, 20].

### Nutrition surveillance system in Uganda, 2015–2018

As per the Uganda Ministry of Health (MoH), a case of wasting in a PLW is defined as a mid-upper arm circumference (MUAC) in the red (< 19.0 cm) or yellow (≥19.0 to < 22.0 cm) zone on the MUAC tape [21]. The MoH captures nutrition data through the Health Management Information System (HMIS). These data are generated from various registers at the key health facility contact points, including integrated nutrition registers, outpatient department registers, antenatal clinic registers (ANC), postnatal clinic registers, immunization/young child clinics, maternity wards, HIV clinics, and in-patient wards and are collated in HMIS form 106a, after which they are entered into DHIS2. The MoH summarizes these data on a monthly, quarterly, and annual basis and disseminates the findings to various stakeholders.

### Study variables and data abstraction

We extracted data from DHIS2 from 2015 to 2018. The variables obtained were PLW assessed for wasting using MUAC, PLW with yellow MUAC, and PLW with red MUAC. We also extracted data on the reporting rates of the HMIS 106a form for the same period to estimate rates of under-reporting.

### Data management and analysis

We extracted nutrition data from DHIS directly into Microsoft Excel for cleaning. We excluded all districts with more malnourished PLW than those seen at ANC from the analysis. We imported the data into Epi-Info version 7 for analysis. We calculated the prevalence of wasting (moderate and severe) at national and regional levels, disaggregated by year. The numerator was all wasted PLW as measured by MUAC. The number of PLW assessed for wasting using MUAC within the analysis period was used as the denominator to calculate prevalence. We used the WHO categorization of wasting by prevalence which considers< 5% acceptable, 5–9% poor, 10–14% serious and ≥ 15% critical to guide interventions [22, 23].. These thresholds are used in decision-making to determine the need for different types of feeding programmes. We generated trends nationally and regionally; drew line graphs to demonstrate the trends; and used logistic regression to test for significance of the trends. We used quantum geographic information system (QGIS) version 2.8.2 to generate maps to demonstrate the geographical distribution of wasting among PLW by region.

## Results

### Prevalence of wasting among pregnant and lactating women, overall and by region, Uganda, 2015–2018

**Fig. 1:**
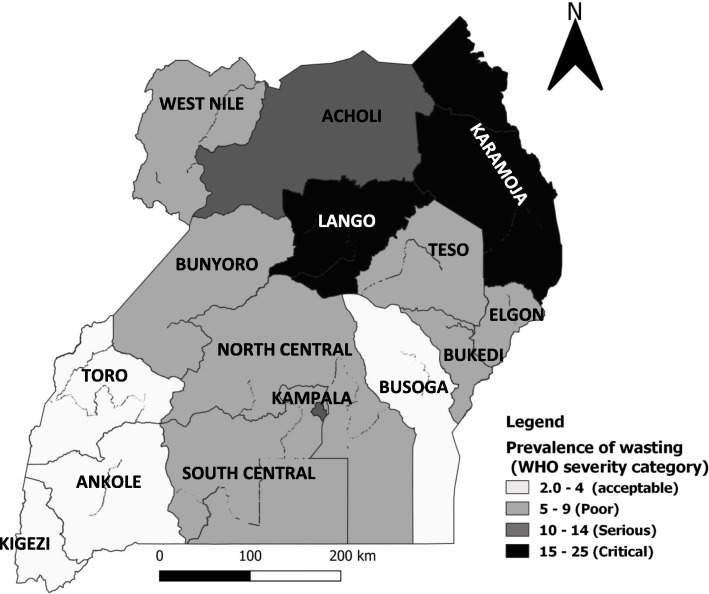
Prevalence of wasting among pregnant and lactating women, Uganda, 2015–2018

Of the 4,848,873 pregnant and lactating women assessed for wasting over the study period, 268,636 (5.5%) were wasted. The median prevalence was 6.8%. Regions in the northern part of the country had the highest overall prevalence of wasting, while those in the regions in the western part had the lowest overall prevalence (Fig. 1).

According to the WHO classification of malnutrition, Karamoja and Lango were in the critical malnutrition category (prevalence > 15%) while Acholi was in the serious malnutrition category (Fig. 1).

### Trends of wasting among pregnant and lactating women, overall and by region, Uganda, 2015–2018

The national annual prevalence of wasting among pregnant and lactating women declined by 31% from 2015 to 2018 (OR = 0.69, *p* < 0.001) (Table 1). The district reporting rate for nutrition was nearly complete across all years (98–100%).

**Table 1:** Regional trends in prevalence of malnutrition among pregnant and lactating women, Uganda 2015–2018

Region	Prevalence (%)				OR (95% CI)
	2015	2016	2017	2018	
**Acholi**	1.4	7.3	16	9.6	1.2 (1.2–1.2)
**Ankole**	24	4.5	2.8	1.7	0.48 (0.47–0.49)
**Bukedi**	6.0	1.6	0.8	11	1.4 (1.3–1.4)
**Bunyoro**	7.3	1.3	3.3	6.3	0.95 (0.92–0.97)
**Busoga**	29	0.7	11	6.1	1.3 (1.3–1.3)
**Bugisu**	1.1	0.7	5.6	4.0	3.4 (3.2–3.6)
**Kampala**	77	15	4.1	1.1	0.13 (0.13–0.14)
**Karamoja**	70	5	16	17	0.55 (0.54–0.55)
**Kigezi**	1.5	2.8	3.2	0.6	0.66 (0.65–0.67)
**Lango**	11	14	8.9	28	1.6 (1.6–1.7)
**North Central**	3.1	5.6	5.3	4.0	0.88 (0.86–0.9)
**South Central**	0.9	14	5	5.8	0.74 (0.73–0.75)
**Teso**	6.9	13	6.4	6.2	0.81 (0.80–0.82)
**Toro**	4.2	7.8	1.1	1.6	0.54 (0.53–0.54)
**West Nile**	4.4	7.5	3.4	4.9	0.92 (0.9–0.93)
**National**	**4.5**	**4.9**	**6.4**	**17**	0.69 (0.69–0.69)

**Fig. 2:**
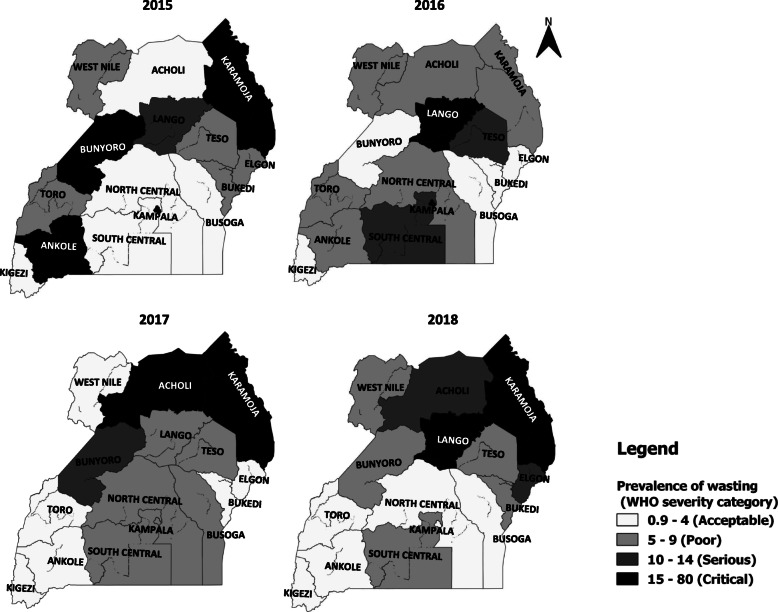
Regional trends in prevalence of wasting among pregnant and lactating women, Uganda 2015–2018

The prevalence of wasting among pregnant and lactating women increased from 2015 to 2018 in Bugisu (OR = 3.4, *p* < 0.001), Lango (OR = 1.6, *p* < 0.001), Bukedi (1.4, p < 0.001), Busoga (OR = 1.3, *p* < 0.001), and Acholi (OR = 1.2, *p* < 0.001) regions, while the rest of the regions had declining trend (Fig. 2).

## Discussion

Analysis of surveillance data from 2015 to 2018 showed a decreasing trend nationally in wasting among pregnant and lactating women. Several regions in the north had increasing trends of wasting, while those in the western and southern parts of Uganda had declined. Two adjoining regions in the north, Karamoja and Lango, had critical levels of malnutrition and a third had serious malnutrition.

Causes of malnutrition are known to be multi-factorial in nature. As a result, strategies such as the Uganda Multi-sectoral Food Security and Nutrition Project (UMFSNP), implemented in 2015 in 15 districts of Uganda [24], have been enacted to address several of these causes jointly. The target population for UMFSNP includes pregnant and lactating women, children under 2 years, and parent groups participating in nutrition-promoting activities. The overall decline in the prevalence of wasting during the study period is likely linked to this approach, at least in part. Regions in which this plan was implemented, including Ankole, Busoga, Kigezi, Tooro, and West Nile, showed declining trends in wasting in this analysis. The possible success of the UMFSNP suggests a need to scale up the implementation of the multisectoral program to additional districts.

Despite the overall decline in the prevalence of wasting countrywide, disaggregated data showed that some regions, especially those in the north, still have high levels of wasting among PLW. Specifically, the northern regions of Karamoja, Lango, and Acholi were found to have the highest prevalence of wasting in this evaluation. These regions have long had prolonged droughts, insecurity, livestock diseases, and flooding which have crippled crop and livestock production [25]; in addition, they consistently experience high levels of poverty and often score poorly across multiple health indicators [13, 26, 27]. Karamoja region is mainly occupied by nomadic pastoralists, who typically do not settle in a single place to cultivate crops. This further deepens the food insecurity and also hinders access to preventive measures and treatment of major illnesses that cause or result from malnutrition [25]. The introduction of modern and innovative farming methods for the northern regions, such as use of drought-resistant seeds and mechanization of farming, could help reduce the food insecurity in these regions and mitigate malnutrition [28].

Despite having the highest prevalence of wasting in our evaluation, the Karamoja region did register a decline, possibly due to the ongoing food aid it has received for over 40 years [29, 30]. While fixed clinics exist in the region, the establishment of supplemental mobile clinics to provide health and nutrition services within Karamoja and surrounding regions might help provide the essential nutrition interventions. This approach has improved health services among other nomadic populations in Kenya and other parts of Africa [31, 32]. Intensification of supplementary and therapeutic feeding programs for pregnant and lactating women is also recommended [23]. Nutrition programs targeting households rather than individuals might improve outcomes, as food in this region is shared within households even when delivered to a single targeted individual, such as a pregnant or lactating mother [33].

Bugisu region, located in eastern Uganda, showed an increase in wasting during the evaluation period, reaching the ‘serious’ severity level in 2018. This region is subject to recurrent natural disasters, such as mudslides, and floods, that cause crop loss; the most recent of these events occurred during December 2019 [34–36]. These types of natural disasters are known to be associated with subsequent malnutrition [37–39]. Implementation of assistance in form of food and/or cash transfers could help in preventing malnutrition in such emergencies and should be evaluated as a possible policy option. Such interventions have helped improve the nutrition outcomes in similar contexts. Studies in multiple countries have shown that cash transfers alone or in combination with other interventions prevented malnutrition and improved outcomes [40–42].

### Study limitation

It is likely that some PLW were counted more than once due to repeat visits to facilities; this may have led to an overestimation of the burden of wasting in our evaluation. Alternatively, the low HMIS reporting rates, especially in 2018, may have led to an underestimation of wasting.

## Conclusion and recommendations

The national trend of wasting among pregnant and lactating women declined during 2015–2018. Lango and the Acholi regions had high and rising rates of wasting. Karamoja and Lango regions experienced critical malnutrition, while Acholi region experienced serious malnutrition. We recommended that the Ministry of Health sustains interventions in place for malnutrition with special attention to Karamoja, Lango, and Acholi regions. A causal analysis to clearly understand the factors underlying malnutrition among PLW in these regions should be conducted and consideration given to implementing alternative approaches to addressing malnutrition in locations with chronically high rates.

## Data Availability

The datasets upon which our findings are based belong to the Uganda Public Health Fellowship Program. For confidentiality reasons, the datasets are not publicly available. However, the data sets can be availed upon reasonable request from the corresponding author and with permission from the Uganda Public Health Fellowship Program.

## Abbreviations

ANC: Antenatal care
CDC: US Centers for Disease Control and Prevention
DHIS2: District Health Information Software version 2
HMIS: Health Management Information System
MoH: Ministry of Health
MUAC: Mid upper arm circumference
OR: Odds ratio
PLW: Pregnant and lactating women
UBOS: Uganda bureau of statistics
UMFSNP: Uganda Multi-sectoral Food Security and Nutrition Project
WHA: World Health Assembly
WHO: World Health Organization
